# The Role of Mouth Infections and Mouth Abnormalties in the Causation of Disease*Address delivered before the Eastern Association of Graduates of the Angle School of Orthodontia, May 5, 1916.

**Published:** 1917-01

**Authors:** Eugene Lyman Fisk

**Affiliations:** Director of Hygiene, Life Extension Institute, New York City


### TEXAS MEDICAL JOURNAL

DR. F. E. DANIEL,
Founder.

Established July,
1885.

MRS. F. E. DANIEL,
Publisher and Managing
Editor.

VOL. XXXII No. 7
AUSTIN,
JANUARY, 1917.

PUBLISHED MONTHLY.
Subscription:
$1.00 a Year.

The publisher is not responsible for views of contributors.

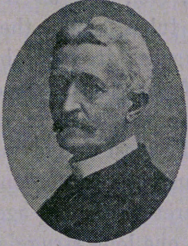

*"All of good the past hath had,
Remains to make our own time glad."*—WHITTIER.

### Original Articles.

### The Role of Mouth Infections and Mouth Abnormalties in the Causation of Disease*

BY EUGENE LYMAN FISK, M. D., DIRECTOR OF HYGIENE, LIFE EXTENSION INSTITUTE, NEW YORK CITY.

There is no more important field of preventive medicine than that of the hygiene of the head. The more one studies the modern man—civilized, cultured, pampered, served by scientifically harnessed natural forces almost as was Aladdin by the slaves of the lamp—the more evident it becomes that disease, old age and death fairly radiate from the cavities of the head.

Take, for example, the repeated acute infections having their origin in obstructed nasal passages lined with unhealthy mucous membranes poorly equipped to meet infection. Season after season the mass of the population suffer from these nasopharyngeal affections involving acute bacterial attack and resultant strain of organic tissue. In some cases death occurs from acute pneumonia, mastoditis, brain abscess, endocarditis; in others, chronic pulmonary disease or tuberculosis.

Setting aside the effects of repeated frank acute nasopharyngeal affections, rhinitis, grippe, tonsilitis, regarded as more or less normal experiences of the average individual, and also eliminating

*Address delivered before the Eastern Association of Graduates of the Angle School of Orthodontia, May 5, 1916.

pneumonia and tuberculosis, we have to consider the effects of the establishment in the various head cavities of chronic foci of infection. These cavities, tooth sockets, accessory sinuses, tonsilar crypts, middle ear, etc., are, as Rosenow and others have shown, veritable culture tubes, where the streptococci and pneumococci go into training, as it were, before streaming out to attack those portions of the body where they can best thrive. Thus it is no mere figure of speech to say that in the head are the training camps for the most dangerous enemies of the human race. These micro-organisms are among the chief causes of chronic disease, and therefore of old age and death. As Mayo has expressed it, "Life is one long struggle with micro-organisms."

Deficiency diseases we undoubtedly have, beri-beri, scurvy and probably pellagra, but I am firmly convinced that long before such stages of serious tissue impairment due to deficiency of vitamins or other necessary elements are reached, there are various degrees of deprivation that lessen the resistance to bacterial attack, sub-standard conditions of the body that pass unnoticed and unnamed until some organ obviously fails in its task, and then we call it disease.

Eliminating the so-called communicable diseases, such as typhoid and tuberculosis, we still have a tremendous death rate from disease that is essentially due to infection. Chronic cardio-renal-vascular diseases are directly responsible for 28 per cent of the total death rate. We know now that in all these organic affections, bacteria play a prominent and often the principal part. Injury, strain, poisons, endogenous or exogenous, all have their influence, but bacterial attack must be recognized at some stage of the game. I believe I am safe in saying that if the cavities in the head could be kept in a thoroughly healthy state, free from infection and their functions well maintained, at least four years could be added to the expectation of human life. This sounds like a little bit, but it means a tremendous reduction in the death rate.

I would not minimize the rôle of the intestine as a great focus of sub-infection and of miscalled auto-intoxication. But it is not as guilty as formerly supposed. Indeed, in much of the damage that it does it is merely an accomplice after the fact of focal head infection that has traveled downward.

The freedom with which bacteria range throughout the human organism has only lately been recognized. Sub-infection, radiating from focal infection, must be recognized as one of the principal factors in organic impairment. Even in tuberculosis we find that the process of infection is not so simple as formerly supposed. The fact that tubercule bacilli can traverse the stomach and intestinal canal, penetrate the walls of the intestine without leaving any trace, and finally reach the bronchial glands or lungs, is an illustration of the losing fight that mankind is maintaining with various forms of bacteria.

Since death comes to all ultimately, always in the form of a pathological state, I am justified in terming this a losing fight. What I have said regarding the hygiene of the head and the importance of the head cavities as potential factors in organic disease emphasizes the tremendous responsibility upon those who are specializing in that region. It is the region par excellence for the entry of infection, as well as for nutritive material and oxygen.

It would be difficult to find an individual with absolutely normal and perfectly functionating head cavities. In using this expression, I am of course excluding the brain. Harry Campbell has recently stated that there are upwards of two hundred million of carious teeth in the British Isles and an even greater number of infected tooth sockets, while as to jaws, it is rare to meet a normal one. There is no reason to think that conditions are any better in this country. Carious teeth, false teeth, long teeth, irregular teeth, infected gums, asymmetric jaws, are the rule rather than the exception. The field, therefore, is an almost limitless one, and the chief responsibility upon the specialist, as upon all others who are working in modern medicine, is to begin as early as possible in the life of the individual to detect abnormalities of these cavities, correct them, and protect the individual from future impairment.

The protection of the deciduous teeth falls to the parent and the dentist, as a part of mouth hygiene. While success in this direction will limit the need for the orthodontist, there will always be plenty for him to do in correcting those forms of irregularity and asymmetry due to heredity or other causes. Also, on the one hand, the orthodontist can lessen the need for rhinological work and septal operations, while, on the other hand, the rhinologist can in his own field assist the individual to a healthy and more vigorous development of the upper jaw, and lessen the need for orthodontia in that region. Also, the general practitioner and hygienist can, by regulating the diet and training the child to eat properly, prevent dental irregularities.

It is desirable to carry to the profession at large the knowledge of the possibilities of orthodontia, knowledge that it performs far more than a cosmetic service, that while the relief of deformities has a very high value from a social and even health standpoint, these tooth irregularities contribute very largely to mouth infection and hence to organic disease. It seems true that the modern mouth is degenerate in its parts.

There may be members present who have studied this question more deeply than I have, in which case I should be more than glad to have additional evidence on this point. There is, however, abundant evidence to show that in a state of nature man's jaws are more vigorous and symmetrically developed, and that he is also comparatively free from other head abnormalities and asymmetries so common among the civilized. Whether early mental development and education strain have anything to do with this matter, as some authorities have suggested, I am unable to say, but heredity and deficiencies in the diet in infancy and childhood, especially those leading to faulty mastication, as well as the long list of hygienic errors that characterize civilized life, must be given due weight.

These possibilities of head infection emphasize what is true of all the other regions of the body, namely, that from infancy on they should be regularly inspected for abnormal condition. Dr. Cabot suggests that these inspections are of limited value unless made by specialists in each region. That is not feasible as applied to the mass of the public, but under a properly standardized and supervised routine, as advocated and operated by the Life Extension Institute, a well-trained general practitioner can make such periodic examinations to detect the need for further special attention.

We must sharply distinguish between the clinic for established disease and its treatment, and this sifting process by which the early signs of disease are noted and the patient shunted to whatever special clinic or clinician his needs may require.

It is amazing what an automatic, systematic sieve of this description will reveal. Indeed, its precise, machine-like, impersonal regularity, applying the same tests in all cases, regardless of the age, health or apparent condition of the individual, constitutes its highest value. In fact, it will often bring out more evidences of impairment than is usually the case when a man drifts aimlessly around from one specialist's office to another. Even a group of specialists trained to consider well-established disease may overlook defects of importance, lying in the intervals between these specialists. I heartily agree with Dr. Cabot as to the great value of the system of group consideration by specialists for therapeutic purposes, after this preliminary though searching survey by the hygienist, at a relatively low cost.

If we have to rely upon specialists to take the blood pressure, examine the arteries, test the reflexes, look into the eye, nose and throat to determine whether there is any abnormality, entirely apart from final diagnosis, what is left for the ordinary physician? The specialist should be drawn from the ranks of the ordinary, generally trained men. The men who lack this general training and ability to survey the body generally, are simply dangerous as specialists. There is a vast supply of these generally trained men, and all they need is a little special instruction and more encouragement to acquire diagnostic technic in the various regions of the body. The examiner who is trained and educated to make a complete health survey performs the functions of a sort of medical airman, who searches out the enemy's weaknesses and gives information by which successful attack can be made by whatever arm of the service will prove most effective, whether it be orthodontia, abdominal surgery or internal medicine

The following figures from the examinations of the Institute will give you an idea of the application of this principle:

RESULTS OF PHYSICAL EXAMINATION OF INDUSTRIAL AND COMMERCIAL WORKERS BY THE LIFE EXTENSION INSTITUTE COMPARED.

| | Industrial Average age 31.6 yr. | Commercial Average age 27.8 yr. |
|---|---|---|
| Free from physical defect or unhealthful living habits | 0.46% | 0.81% |
| Slight impairment or errors in living habits | 27.16% | 22.96% |
| Moderate impairment needing medical, dental or hygienic treatment | 67.15% | 71.91% |
| Important impairment requiring close medical supervision and treatment | 5.20% | 4.29% |

It will interest you to know that 70 per cent of these people examined showed need of dental attention, and that among the number showing that need organic impairment was found to the extent of 24 per cent in excess of those without dental impairment.

I think you will agree with me that this specialized assistance, such as workers in the field of orthodontia are able to render, should not be left to the haphazard initiative of patients. The detection of these abnormalities that require your aid should be a systematic routine affair, applied to the whole population. It is then up to you to provide facilities by which the benefits of this treatment may not be limited to the idle rich, but extended by some means to all who need it.

